# Plastic and genetic responses of a common sedge to warming have contrasting effects on carbon cycle processes

**DOI:** 10.1111/ele.13178

**Published:** 2018-11-22

**Authors:** Tom W. N. Walker, Wolfram Weckwerth, Luca Bragazza, Lena Fragner, Brian G. Forde, Nicholas J. Ostle, Constant Signarbieux, Xiaoliang Sun, Susan E. Ward, Richard D. Bardgett

**Affiliations:** ^1^ School of Earth and Environmental Sciences The University of Manchester Manchester M13 9PL UK; ^2^ Centre for Ecology and Hydrology Lancaster LA1 4AP UK; ^3^ Lancaster Environment Centre Lancaster University LA1 4YQ Lancaster UK; ^4^ Department of Ecogenomics & Systems Biology University of Vienna 1090 Vienna Austria; ^5^ Vienna Metabolomics Centre (VIME) University of Vienna 1090 Vienna Austria; ^6^ Swiss Federal Institute for Forest Snow and Landscape Research (WSL) 1015 Lausanne Switzerland; ^7^ Ecological Systems Laboratory (ECOS) École Polytechnique Fédérale de Lausanne (EPFL) 1015 Lausanne Switzerland; ^8^ Department of Life Science and Biotechnologies University of Ferrara 44100 Ferrara Italy

**Keywords:** Carbon cycle, climate feedbacks, climate warming, *Eriophorum vaginatum*, genetic adaptation, intraspecific variation, natural selection, phenotypic plasticity, plant ecophysiology, plant metabolism

## Abstract

Climate warming affects plant physiology through genetic adaptation and phenotypic plasticity, but little is known about how these mechanisms influence ecosystem processes. We used three elevation gradients and a reciprocal transplant experiment to show that temperature causes genetic change in the sedge *Eriophorum vaginatum*. We demonstrate that plants originating from warmer climate produce fewer secondary compounds, grow faster and accelerate carbon dioxide (CO_2_) release to the atmosphere. However, warmer climate also caused plasticity in *E. vaginatum*, inhibiting nitrogen metabolism, photosynthesis and growth and slowing CO_2_ release into the atmosphere. Genetic differentiation and plasticity in *E. vaginatum* thus had opposing effects on CO_2_ fluxes, suggesting that warming over many generations may buffer, or reverse, the short‐term influence of this species over carbon cycle processes. Our findings demonstrate the capacity for plant evolution to impact ecosystem processes, and reveal a further mechanism through which plants will shape ecosystem responses to climate change.

## Introduction

Climate warming over the past century has altered the distribution and physiology of plant species in most biomes across the globe (Parmesan & Yohe 2003). Warming‐induced range shifts have been documented for many species (Chen *et al*. 2011), influencing both plant community composition (Alexander *et al*. 2015) and the functioning of ecosystems (Elmendorf *et al*. 2012). It is well established that warming alters the physiology of individual plants over timescales of hours to seasons through phenotypic plasticity (Nicotra *et al*. 2010). Additionally, sustained warming can lead to natural selection in plant populations, driving genetic adaptation and changes to phenotypes over multiple generations (Hairston *et al*. 2005; Hancock *et al*. 2011; Merilä & Hendry 2014; Siepielski *et al*. 2017). Phenotypic plasticity and genetic adaptation occur in concert and both can influence plant tissue composition (Anderson & Gezon 2015), metabolism (Scherling *et al*. 2010), photosynthesis (Albert *et al*. 2011), phenology (Anderson *et al*. 2012) and growth (Kim & Donohue 2013). However, phenotypes generated by plasticity may or may not be representative of those arising through adaptation (Ghalambor *et al*. 2015; Becklin *et al*. 2016), and the relative importance of plasticity and adaptation in dictating ecosystem responses to warming is unknown.

Quantifying the responses of plants to warming is central for understanding the global carbon cycle and its feedbacks to future climate change (Ostle *et al*. 2009). Plants underpin the carbon cycle, both as the source of photosynthetic carbon for ecosystems and a key regulator of carbon dioxide (CO_2_) release from ecosystems to the atmosphere. Changes in plant tissue composition (Cornwell *et al*. 2008), net primary production (Ward *et al*. 2013), growth (Elmendorf *et al*. 2012) and root and litter inputs (Fontaine *et al*. 2007) are known to influence carbon cycling *via* their control over the activity and composition of soil‐dwelling microorganisms (Metcalfe *et al*. 2011). In turn, responses of soil microbes to vegetation may cause feedbacks to plant growth and community dynamics (Lau & Lennon 2011), shaping plant assemblages and carbon cycle processes (Bardgett *et al*. 2013). Most research exploring the role of plants in carbon cycling has done so at the community or species scale. However, intraspecific variation accounts for more than 25% of plant community variation worldwide (Siefert *et al*. 2015), and there is emerging evidence that it may be an important driver of soil microbial community composition (Bailey *et al*. 2009), organic matter decomposition (Schweitzer *et al*. 2005) and greenhouse gas emissions (Fischer *et al*. 2014). Plant responses to warming at the population scale thus have the capacity to influence carbon cycling at the ecosystem scale. Despite the potential for this to feed back to the Earth's climate system, the consequences of warming‐induced intraspecific variation for carbon cycling remain unexplored.

Environmental gradients provide a powerful tool for detecting natural selection in non‐model species (White *et al*. 2013; Merilä & Hendry 2014). By quantifying genetic mutations (DNA polymorphisms) common to multiple populations on a gradient, it is possible to identify those that change in frequency with the major axis of environmental change. Polymorphisms with a higher frequency at one end of the gradient are likely to be associated with alleles that confer a selective advantage to individuals in that environment, or alternatively a selective disadvantage in the environment at the other end of the gradient (White *et al*. 2013; Merilä & Hendry 2014). If consistent across multiple gradients and large geographical areas, such polymorphisms are evidence that natural selection occurs due to the environmental change common to all gradients (Hancock *et al*. 2011; White *et al*. 2013; Alonso‐Blanco *et al*. 2016). Coupling gradients with reciprocal transplant experiments facilitates robust discrimination between the traits immediately responsive to environmental change (i.e. phenotypic plasticity) and those arising due to genetic change over multiple generations (e.g. genetic adaptation; Blanquart *et al*. 2013; Merilä & Hendry 2014).

**Figure 1:**
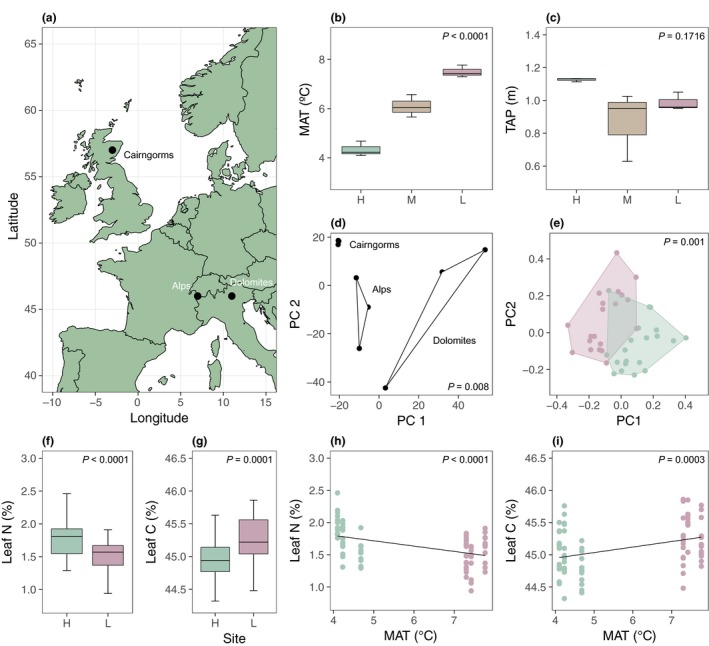
Locations and characteristics of elevation gradients. (a) Elevation gradients were established in three European mountain areas supporting populations of *E. vaginatum*. (b and c) Boxplots showing (b) mean annual temperature (MAT; °C) and (c) total annual precipitation (TAP; cm) for high (H, high), mid (M, orange) and low (L, red) elevation sites. (d) PCs 1 and 2 from a PCA of SNP allele frequencies grouped by mountain area. (e) PCs 1 and 2 from a PCA of plant phenotypes for high (green) and low (red) elevation populations on one gradient. (f and g) Boxplots showing (f) leaf nitrogen and (g) leaf carbon contents (%) for high (green) and low (red) elevation populations in all mountain areas. (h and i) Scatter plots showing (h) leaf nitrogen and (i) leaf carbon (%) against (h) MAT (°C) and (i) TAP (cm) using high (green) and low (red) elevation populations from all mountain areas. For (b–d), *P*‐values indicate significance of differences between displayed groups (see Appendix S5).

Our aim was to determine whether climate warming causes intraspecific variation in the ubiquitous sedge *Eriophorum vaginatum* L. that has consequences for carbon cycle processes. *E. vaginatum* is a slow‐growing perennial sedge that is likely to react slowly to climate change (Wein 1973). It is found in cold northern biomes, which are vulnerable to rapid warming (IPCC 2013), and plays an important role in the carbon cycle by accelerating both plant (Trinder *et al*. 2008; Ward *et al*. 2009) and soil (Walker *et al*. 2016) respiration. We thus used *E. vaginatum* as a conservative model species for assessing the importance of warming‐induced plant intraspecific variation for the carbon cycle. Our approach was twofold. First, we used three elevation gradients in geographically distinct mountain regions of Europe (Fig. 1a) to test the hypothesis that genetic change in *E. vaginatum* (i.e. putative natural selection) is related to temperature. Elevation gradients are widely used to test for the role of climatic factors, especially temperature, on plants and ecosystem processes (Sundqvist *et al*. 2013; Graham *et al*. 2014; Mayor *et al*. 2017). Their use here enabled us to test whether genetic change in *E. vaginatum* populations was explained by temperature, as opposed to other climatic factors (e.g. precipitation or UV radiation). Second, we established an independent reciprocal transplant experiment on one of the elevation gradients to test two hypotheses: (1) *E. vaginatum* populations adapted to warmer climate possess different traits to those adapted to cooler climate, resulting in different effects on carbon cycle processes; and (2) exposure to warmer climate induces phenotypic plasticity in *E. vaginatum* traits, also with consequences for carbon cycle processes. We specifically quantified shifts in phenotypes on the molecular (primary and secondary metabolisms), leaf (tissue composition, photosynthetic investment) and whole‐plant (growth) scales, alongside local rates of ecosystem respiration, gross photosynthesis and net CO_2_ exchange. Reciprocal transplant experiments between high and low elevation sites have been extensively used to test for the direct effects of temperature on plants and ecosystems (e.g. Anderson *et al*. 2012; Kim & Donohue 2013; Alexander *et al*. 2015). Here, by linking a reciprocal transplant experiment to a population genetics approach on multiple elevation gradients, we were able to not only quantify temperature effects on plant phenotypes and the carbon cycle, but also to provide our findings with a potential evolutionary mechanism.

## Materials and methods

### Study sites and experimental design

We established elevation gradients (Fig. 1a) in the Scottish Cairngorms (57°06′N, 03°20′W), the Swiss Alps (46°40′N, 07°40′E; 46°34′N, 06°10′E) and the Italian Alps/Dolomites (46°25′N, 11°24′E). We selected low, medium and high elevation sites at each gradient that were typical of treeless northern peatlands and supported populations of *E. vaginatum*. Northern peatlands are globally consistent in soil type (i.e. peat), moisture (i.e. water table at the surface) and pH (i.e. acidic) (Moore 2002). Peatlands are also functionally consistent within Europe in vegetation composition (Robroek *et al*. 2017), with *E. vaginatum* being the dominant graminoid species. While specific elevations varied between mountain areas (see Appendix S1 in Supporting Information), they represented similar gradients of temperature change (Fig. 1b,c) and captured the full *E. vaginatum* elevational range in each region. The use of three geographically distinct gradients thus enabled us to identify elevation‐induced environmental change common to all gradients, and to determine its influence over genetic change in *E. vaginatum*. Temperature (mean annual temperature, MAT; °C) and precipitation (total annual precipitation, TAP; cm) were monitored using data provided by local meteorological stations (Cairngorms: MIDAS; Dolomites: Ufficio Idrografico, Bolzano, Ufficio Previsioni e Pianificazione, Trento; Switzerland: Meteo Suisse) for the period 2004 to 2014.

### Assessing elevation‐induced genetic change: population genetics

We sampled leaf tissue from 50 *E. vaginatum* individuals at each site/gradient for population genetics in July 2012 (*N* = 450; minimum 7 m between sampled plants). Tissue was dried immediately in silica gel and stored in the dark until DNA extraction. Desiccated leaf tissue was milled (MM400; Retsch, Han, Germany) and 5 mg of powder from each plant was pooled into one of nine composite population samples (i.e. one sample for each site; *N* = 9). We made five technical replicates of the weighing, pooling and DNA extraction steps to minimise error caused by unequal representation of individuals in population samples (*N* = 45). While such an approach could not entirely eliminate this source of error, pooling biases have been shown to be unimportant in population genetics analyses (Emerson *et al*. 2010; Gautier *et al*. 2013; Schlötterer *et al*. 2014; Hivert *et al*. 2018), and in this case would have reduced, not increased, the likelihood of detecting significant elevation‐induced SNP allele frequency changes. Genomic DNA was extracted (DNEasy Plant Maxi Kit; Qiagen, Venlo, Netherlands), cleaned (Crouse & Amorese 1996) and purified (50 ng μl^−1^, A_260_/A_280_ = 1.75–1.95), following which we pooled technical replicates into nine final samples and sent them to Edinburgh Genomics, The University of Edinburgh, UK, for restriction site‐associated DNA sequencing and data preparation (Davey *et al*. 2011) (see Appendix S2). We labelled resulting DNA sequence reads that differed in sequence by one nucleotide as single nucleotide polymorphisms (SNPs) and calculated an expected allele frequency for each SNP as the probability observing one allele within a population (Davey *et al*. 2011). We retained SNPs with a depth of coverage between 10 and 10 000 (Gautier *et al*. 2013) and a minor allele frequency of greater than 10% across all populations (White *et al*. 2013). Finally, we discarded loci with allele frequencies of between 0.4 and 0.6 to filter out potential paralogs (i.e. gene duplicates) with single nucleotide differences, which if retained could falsely be interpreted as SNPs (White *et al*. 2013).

### Assessing climate effects on plant phenotypes: reciprocal transplant experiment

#### Experimental design

We established a 2‐year reciprocal transplant experiment in June 2013 between the high and low elevation sites of the Swiss Alps gradient as the best characterised gradient in terms of vegetation composition and soil properties (Bragazza *et al*. 2012). The sites were homogenous in slope, microtopography, soil temperature and soil moisture (data not shown). We selected 20 adult *E. vaginatum* individuals from each elevation (i.e. source population) from representative locations across the sites, maintaining a minimum distance of 7 m between plants. We removed plants with roots intact, rinsed the roots in stream water, replanted 10 at the home site and transplanted 10 to the other elevation site. Disturbance caused no mortality over the 2‐year study period and had no detectable effects on *E. vaginatum* physiology when compared to an undisturbed control group (see Appendix S3).

#### Molecular scale: metabolomics

We measured concentrations of leaf primary and secondary metabolites in July 2014 using a combined extraction and analysis platform with gas chromatography and liquid chromatography coupled to mass spectrometry (GC‐MS; LC‐MS). Five fully emerged green leaves were taken from five individuals per source population at both sites, immediately snap frozen in liquid nitrogen, stored on dry‐ice and freeze‐dried on return to the laboratory. We analysed primary metabolites using GC‐MS (Weckwerth *et al*. 2004) and secondary metabolites using LC‐MS (Scherling *et al*. 2010), including the generation of elemental compositions and quasi van Krevelen plots (Doerfler *et al*. 2014; see Appendix S2). Primary metabolites were mapped to compound classes and biochemical pathways using MapMan (www.mapman.gabipd.org), and all metabolite concentrations were standardised prior to analysis.

#### Leaf scale: photosynthesis, tissue composition

We measured maximum leaf‐level photosynthesis (photosynthetic capacity, *A*_max_; μmol CO_2_ m^−2^ s^−1^) and stomatal conductance (*g*_s_; μmol H_2_O m^−2^ s^−1^) in the field under optimum conditions, alongside maximum PSII efficiency (F_v_/F_m_), specific leaf area (SLA; cm^−2^ g^−1^) and leaf carbon (C) and nitrogen (N) contents (%). Measurements were taken on five fully emerged green leaves from four individuals per source population at both sites in August 2014. We measured *A*_max_ and *g*_*s*_ using an open infrared gas analyser connected to a 2.5 cm^2^ chamber (CIRAS‐2 & PLC‐6, PP Systems, Amesbury, USA). Source population effects on A_*max*_ and g_*s*_ were determined within each site, using measurements taken at the site's ambient partial pressure of CO_2_ (P_CO2_). Elevation effects on A_*max*_ and g_*s*_ were determined for each source population separately using measurements taken at the P_CO2_ of a population's home elevation (i.e. eliminating artefacts generated by changes in atmospheric pressure). Measurements were corrected using measurements of specific leaf area (SLA; cm^−2^ g^−1^) on the same leaf tissue, and tissue was also used to determine leaf C and N contents (%). Atmospheric pressure had no current or legacy effects on leaf C content, in that we observed no change to leaf C between planting elevations (LR_1,14_ = 1.42, *P *=* *0.2328) or source populations (LR_1,14_ = 0.85, *P *=* *0.3558). We quantified leaf maximum PSII efficiency (F_v_/F_m_) with measurements of leaf chlorophyll *a* fluorescence on dark‐adapted (30 mins) leaves from the same plants (PAM‐2500 pulse amplitude fluorometer & 2030B leaf‐clip holders, Heinz Walz, Effeltrich, Germany). Finally, leaf C and N contents were determined for plants situated at high, mid and low elevations of all mountain gradients, using tissue collected for population genetics.

#### Plant scale: growth

We calculated aboveground biomass production (growth; mg d^−1^) on all plants (i.e. *n* = 10 per source population at each site) between the start (June) and end (August) of the 2014 growing season (i.e. 1 year after planting). To do so, we took measurements of maximum tussock height (mm) and diameter (mm) and destructive samples of aboveground biomass (mg) from plants outside the measurement area (*n* = 40) and used a generalised least squares (GLS) model explaining biomass as a function of height and diameter (height × diameter: LR_1,5_ = 23.85, *P *<* *0.0001) to predict the aboveground biomass of focal plants.

#### Carbon cycle consequences: CO_2_ fluxes

We measured local rates of net CO_2_ exchange (NEE; mg CO_2_ m^−2^ h^−1^) and ecosystem respiration (ER; mg CO_2_ m^−2^ h^−1^) on all plants (i.e. *n* = 10 per population per site). Fluxes were taken on multiple dates at each site between June and August 2014. We enclosed plants and underlying soil in airtight chambers (*h* = 32 cm, *d* = 10 cm), using a transparent chamber for NEE and an opaque chamber for ER, and monitored the change in CO_2_ concentration over a 120 s period with an infrared gas analyser (EGM‐4, PP Systems, Amesbury, USA). Soil temperature, soil moisture and photosynthetically active radiation (PAR) were recorded for each measurement and were used alongside measurements of air temperature, enclosure volume and soil surface area to calculate CO_2_ fluxes (Ward *et al*. 2013). We calculated gross photosynthesis (GPP; mg CO_2_ m^−2^ h^−1^) as the difference between NEE and ER. Plant aboveground biomass had no observable effect on CO_2_ fluxes (data not shown).

### Statistical analysis

#### Climate‐induced genetic change

We calculated mean genetic differentiation (*F*_st_) among high, mid and low elevation *E. vaginatum* populations within and between mountain gradients using PoPoolation 2 (Kofler *et al*. 2011). We also quantified genetic variation between mountain gradients using a permanova by Euclidean distance, including all SNP allele frequencies. We combined multiple approaches across all gradients to explore whether directional changes in elevation and/or climate could be responsible for genetic change between *E. vaginatum* populations. We used pairwise Cochran–Mantel–Haenszel tests to determine whether SNP allele frequencies differed between high‐mid, high‐low and mid‐low populations (PoPoolation 2) (Kofler *et al*. 2011), using gradient as a replicate (*n* = 3). We scrutinised the significance of observed *P*‐values using plots of *P*‐value frequencies against local and overall false discovery rates (Storey 2003) and observed vs. expected log_10_‐transformed *P*‐values (see Appendix S4). We also performed within‐gradient correlations between SNP allele frequencies and elevation, MAT and TAP, calculated the mean correlation coefficients across gradients and derived an empirical *P*‐value for each SNP as the ranked mean correlation coefficient divided by the total number of SNPs (White *et al*. 2013). We used the number of SNPs that significantly (empirical *P *<* *0.05) changed in frequency with elevation, temperature and/or precipitation to determine the strength of the relationships between these variables and genetic variation in *E. vaginatum* (Hancock *et al*. 2011; Alonso‐Blanco *et al*. 2016). Finally, we used RDAs on data from the reciprocal transplant experiment to test whether phenotypic variation in *E. vaginatum* populations had a genetic basis and to explore the potential for local adaptation to home vs. away sites (Merilä & Hendry 2014; Monroe *et al*. 2018) (see Appendix S2).

#### Climate effects on plant phenotypes

We tested for effects of source population (i.e. genetic adaptation), planting elevation (i.e. phenotypic plasticity) and their interaction on all traits using standardised GLS models, linear mixed effects (LME) models and permanova by Euclidean distance (see Appendix S2). We found no significant source population × elevation interactions for any response variable (see Appendix S5), meaning that differences between source populations were consistent at both planting elevations and that both populations responded similarly to short‐term elevation change. GLS and LME model outputs were visualised using standardised effects sizes ± 95% confidence intervals, with a positive effect size representing an increase in the response from cool to warm source populations or planting elevations.

#### Climate and plant variation across gradients

We characterised elevation effects on temperature (MAT), precipitation (TAP) and global radiation across all mountain gradients using standardised GLS models. We also explored environment–phenotype relationships at the same spatial scale by performing GLS models between elevation or MAT and leaf C or N contents using measurements from high and low elevation populations at all mountain gradients.

## Results

### Climate variation across gradients

Climate during both study years was typical in terms of MAT and TAP (see Appendix S7). Mountain gradients represented an elevation‐based change in MAT (Fig. 1b; LR_1,5_ = 28.71, *P *<* *0.0001) that was consistent between gradients (gradient: LR_2,5_ = 1.77, *P *=* *0.4133; elevation × gradient: LR_2,3_ = 3.82, *P *=* *0.1482). MAT across all gradients was 4.34 ± 0.18 °C at high elevation and 7.50 ± 0.14 °C at low elevation for the period 2004 to 2014. During the 2‐year study period, MAT at the reciprocal transplant experiment was 4.68 ± 0.24 °C at high elevation and 7.42 ± 0.42 °C at low elevation. By contrast, TAP (Fig. 1c) and solar radiation (see Appendix S6) did not change consistently between elevations (TAP: LR_1,5_ = 1.87, *P *=* *0.1716; radiation: LR_1,34_ = 1.27, *P *=* *0.2589) or gradients (TAP: LR_2,5_ = 2.52, *P *=* *0.2837; radiation: LR_2,34_ = 2.26, *P *=* *0.3230), although solar radiation had gradient‐specific effects (elevation × gradient: LR_2,32_ = 34.80, *P *<* *0.0001).

### Climate‐induced genetic change and putative genetic adaptation

*E. vaginatum* populations situated on different elevation gradients were geographically (Fig. 1a) and genetically distinct (Fig. 1d; *F*_2,6_ = 1.97, *P *=* *0.008; mean ± SE within‐gradient *F*_ST_ = 0.07 ± 0.001; mean ± SE between‐gradient *F*_ST_ = 0.11± 0.001). Pairwise comparisons between high, mid and low elevation populations showed that elevation change was associated with consistent genetic change between populations across all gradients (see Appendix S4). We identified 2808 SNPs in the *E. vaginatum* genome that were present in every population. Of these SNPs, 140 possessed alleles that significantly changed in frequency with elevation in all three mountain areas (empirical *P *<* *0.05), with 77 alleles (55%) also correlating with mean annual temperature and only four alleles (5%) also correlating with total annual precipitation. Using the reciprocal transplant experiment, we found that plant phenotypes, as represented by a matrix of all plant traits, differed significantly between source populations (Fig. 1e; *F*_1,37_ = 11.66, *P *=* *0.001, 17.6% variance) after accounting for significant planting elevation effects (F_1,38_ = 12.10, *P *=* *0.001, 23.9% variance). We also found that phenotypes differed significantly between home and away sites (*F*_1,36_ = 3.08, *P *=* *0.007, 3.2% variance) after accounting for planting elevation and source population effects.

### Reciprocal transplant: source population effects on phenotypes and CO_2_ fluxes

**Figure 2:**
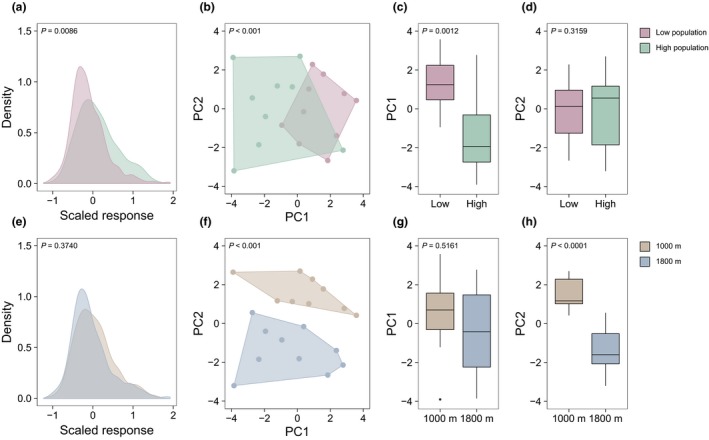
Warming effects on *E. vaginatum* primary metabolism. Effects of (a–d) source population (green, high elevation population; purple, low elevation population) and (e–h) planting elevation (brown, 1000 m; blue, 1800 m) on (a and e) the activity of the primary metabolism (density plots of all standardised peak areas), (b and f) variation within the primary metabolism (plots of PCs 1 and 2 from a PCA considering all primary metabolites), (c and g) variation within PC1 and (d and h) variation within PC2. In all cases, *P*‐values indicate significance of differences between displayed groups (see Appendix S5).

**Figure 3:**
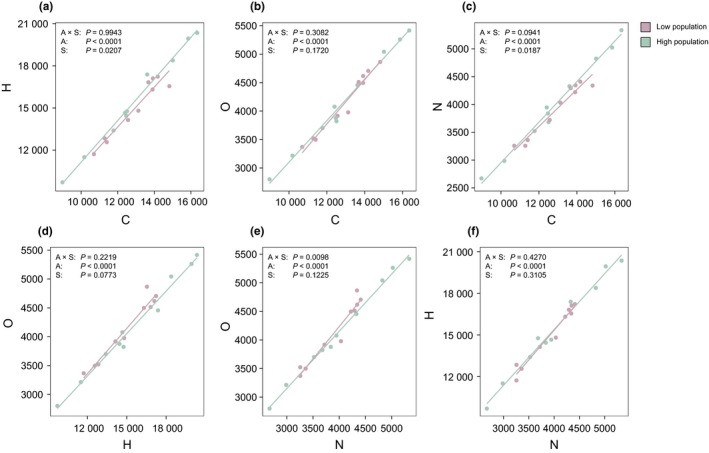
Source population effects on the *E. vaginatum* secondary metabolism. Effects of source population (green, low population; red, high population) on the abundances (atom counts) of carbon (C), hydrogen (H), nitrogen (N) and oxygen (O) atoms in secondary metabolites (*n* = 5; Methods). We determined the significance (*P *<* *0.05) of relationships between atoms (A), source populations (S) and their interaction (A × S) using linear mixed effects models, including planting elevation as a random intercept term. All atom counts were significantly positively correlated (a–f). Some atom counts additionally differed between source populations (a,c and e), demonstrating that high and low source populations invested differently in the secondary metabolism.

**Figure 4:**
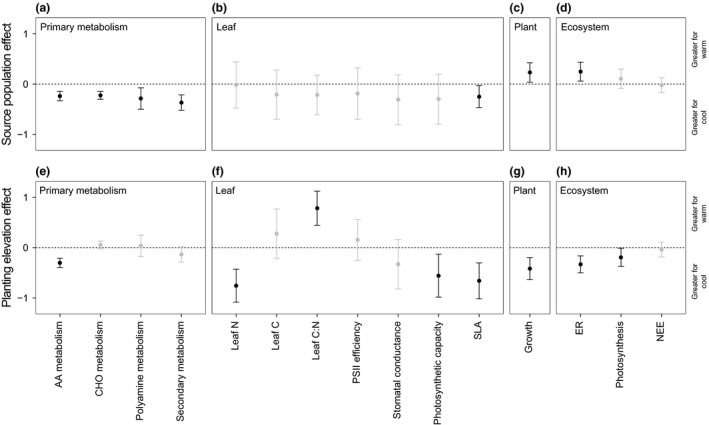
Warming effects on *E. vaginatum* and ecosystem CO_2_ fluxes. Effects of (a–d) genetic differentiation (i.e. source population) and (e–h) phenotypic plasticity (i.e. planting elevation) on *E. vaginatum* phenotypes at the (a and e) molecular scale (amino acid (AA) metabolism; carbohydrate (CHO) metabolism; polyamine metabolism; secondary compound synthesis), (b and f) leaf scale (leaf nitrogen (N) content; leaf carbon (C) content; leaf C:N; maxmimum PSII efficiency (F_v_/F_m_); stomatal conductance; photosynthetic capacity; specific leaf area (SLA)), (c and g) plant scale (growth) and (d and h) ecosystem scale (ecosystem respiration (ER), gross photosynthesis and net ecosystem CO_2_ exchange (NEE)). Responses represent standardised effect sizes of GLS models ± 1 SE, with points situated above or below zero (dotted line) indicating an increase or decrease from high to low population/elevation, respectively. Responses with error bars not intersecting zero (black) are significant (*P *<* *0.05), and non‐significant responses are shaded grey (statistical test outputs are given in Appendix S5).

Plants from the low elevation population had a primary metabolism that was both less active than (Fig. 2a; LR_1,17_ = 6.90, *P *=* *0.0086) and functionally distinct (Fig. 2b; *F*_1,16_ = 2.79, *P *=* *0.002) from that of plants from the high elevation population. Specifically, the low elevation population had lower concentrations of 23 out of 82 quantified primary metabolites (see Appendix S8), and invested significantly less in polyamine metabolism (e.g. nicotinic acid, spermidine; LR_1,341_ = 6.94, *P *=* *0.0084) and secondary metabolite synthesis (e.g. caffeoylquinic acid, chlorogenic acid, shikimic acid; LR_1,341_ = 21.03, *P *<* *0.0001) than the high elevation population (Fig. 4a). Moreover, plants from the low elevation population invested differently in secondary metabolism overall (Fig. 3; H:C: LR_1,16_ = 5.35, *P *=* *0.0207; N:C: LR_1,16_ = 5.54, *P *=* *0.0187). Metabolic differences occurred irrespective of the environment experienced by plants during the 2‐year study period, and explained more overall variation in primary metabolism (i.e. PC 1, 24.6% explained variance) than planting elevation (Fig. 2c; LR_1,17_ = 10.35, *P *=* *0.0013; vs. Fig. 2g; LR_1,17_ = 0.42, *P *=* *0.5161). We found no differences in leaf N content (LR_1,14_ = 0.01, *P *=* *0.9337), C content (LR_1,14_ = 0.85, *P *=* *0.3558), PSII efficiency (LR_1,14_ = 0.03, *P *=* *0.8607), stomatal conductance (LR_1,12_ = 1.73, *P *=* *0.1890) or photosynthetic capacity (LR_1,12_ = 1.62, *P *=* *0.2024) between the two populations (Fig. 4b). However, SLA was ~10.5% lower in the low source population (LR_1,14_ = 14.84, *P *=* *0.0271; Fig. 4b). Despite this, plants from the low elevation population grew 2.3 times faster than plants from the high elevation population at both planting elevations (Fig. 4c; LR_1,37_ = 5.28, *P *=* *0.0126), and increased rates of ecosystem respiration by 21.6% (Fig. 4d; LR_1,72_ = 6.30, *P *=* *0.0120). Rates of gross photosynthesis and net CO_2_ exchange did not differ significantly between source populations (Fig. 4d; GPP: LR_1,71_ = 1.18, *P *=* *0.2772; NEE: LR_1,71_ = 0.09, *P *=* *0.7612).

### Reciprocal transplant: planting elevation effects on phenotypes and CO_2_ fluxes

Plants located at different elevations had primary metabolisms that were similarly active (Fig. 2e; LR_1,17_ = 0.79, *P *=* *0.3740), but functionally distinct (Fig. 2f; F_1,16_ = 3.09, *P *=* *0.002). Plants situated at the low elevation site possessed lower amino acid concentrations than plants at the high elevation site (e.g. glutamic acid, glycine, isoleucine, leucine, pyroglutamic acid, serine, threonine and valine; see Appendix S8) and displayed a reduced amino acid metabolism overall (Fig. 4e; LR_1,341_ = 39.32, *P *<* *0.0001). At the same time, plants at the low elevation site had higher concentrations of oxoglutaric acid and some carbohydrates than plants at the high elevation site (see Appendix S8). Metabolic differences between planting elevations occurred in plants from both populations and, while less important than differences caused by source population (i.e. PC2, 16.0% explained variance), explained the second most important axis of variation in the primary metabolism (Fig. 2f; LR_1,17_ = 22.89, *P *<* *0.0001). At the leaf level (Fig. 4f), plants located at the low elevation site had significantly lower N content (LR_1,14_ = 9.77, *P *=* *0.0018), photosynthetic capacity (LR_1,12_ = 6.03, *P *=* *0.0140) and SLA (LR_1,14_ = 19.22, *P *=* *0.0023) than plants at the high elevation site, but had similar C content (LR_1,14_ = 1.42, *P *=* *0.2328), PSII efficiency (LR_1,14_ = 0.13, *P *=* *0.7166) and stomatal conductance (LR_1,12_ = 1.96, *P *=* *0.1610). Moreover, plants at the low elevation site grew 15.0 times slower (Fig. 4g; LR_1,37_ = 11.19, *P *=* *0.0008) and photosynthesised 18.7% less (Fig. 4h; LR_1,71_ = 4.37, *P *=* *0.0365) than plants at the high elevation site, irrespective of source population, and promoted 25.3% slower rates of ecosystem respiration (Fig. 4h; LR_1,72_ = 13.57, *P *=* *0.0002). Net CO_2_ exchange did not differ significantly between planting elevations (Fig. 4h; LR_1,71_ = 0.28, *P *=* *0.5985).

### Trait variation across gradients

Across all gradients, low elevation populations possessed 0.25 ± 0.08% less leaf N (Fig. 1f; LR = 18.41_1,82_, *P *<* *0.0001) and 0.30 ± 0.10% more leaf C (Fig. 1g; LR_1,82_ = 15.51, *P *=* *0.0001) than high elevation populations. We also found that higher MAT was generally associated with lower leaf N contents (Fig. 1h; LR_1,82_ = 21.48, *P *<* *0.0001) and higher leaf C contents (Fig. 1i; LR_1,82_ = 13.19, *P *=* *0.0003).

## Discussion

Our aim was to explore whether climate warming drives intraspecific variation in the common sedge *E. vaginatum* that impacts carbon cycle processes. We found that temperature caused genetic differentiation in this species, and that plants originating from warmer climate produced fewer secondary compounds, grew faster and accelerated rates of ecosystem respiration. However, warmer climate also caused plasticity in *E. vaginatum*, lowering investment in nitrogen metabolism, photosynthesis and growth and dampening rates of ecosystem respiration. We caution that we focussed on one species at a single stage of its life cycle, and our transplant experiment was undertaken on only one gradient. Nevertheless, this is the first demonstration that plastic or genetic responses of any plant species to warming have the capacity to affect rates of ecosystem‐level processes involved in the carbon cycle.

Our findings suggest that climate, and predominantly temperature, likely causes natural selection and genetic adaptation in *E. vaginatum*. SNP allele frequencies differed significantly between high, mid and low elevation populations from all mountain areas (see Appendix S4), and 55% of allele frequency changes also correlated with temperature (compared to 5% with precipitation). It is important to note that correlations between temperature and genetic change are only suggestive of natural selection. It is also possible that other factors not measured here, such as soil nutrient availability and plant competitive interactions, which are known to vary along elevation gradients (Bragazza *et al*. 2012; Alexander *et al*. 2015; Mayor *et al*. 2017), have the potential to drive selection in plant populations. Nevertheless, variation in such factors is usually itself a response to temperature (Bragazza *et al*. 2012; Alexander *et al*. 2015), and we found no evidence for alternative gradient‐wide relationships between elevation and other climatic variables, such as precipitation and solar radiation (Fig. 1c; Appendix S6). Given this, and our finding that a significant proportion of SNP alleles in *E. vaginatum* populations correlated significantly with mean annual temperature across three elevation gradients in distinct geographical mountain regions, we suggest that temperature is a dominant factor explaining elevation‐induced genetic change in this species (Hancock *et al*. 2011; Merilä & Hendry 2014; Alonso‐Blanco *et al*. 2016). Further research, however, is needed to determine whether temperature causes genetic differentiation in this species directly, or alternatively indirectly *via* its influence over other ecosystem properties.

Genetic differentiation in *E. vaginatum* was greatest between the Cairngorms in Scotland and other mountain areas (Fig. 1d), which was likely due to the isolation of this region following the last Ice Age (Hewitt 2000). Elevation was associated with genetic change despite such differentiation, and was not the product of genetic history owing to the low differentiation observed between populations overall (mean ± SE *F*_ST_ = 0.10 ± 0.001). We found that genetic differentiation was not only present between high and low elevation populations, but also explained differences in plant phenotypes (Fig. 1e). Moreover, we found that both populations displayed consistent phenotypes at their home sites, suggesting that populations may be locally adapted to specific temperature regimes (Blanquart *et al*. 2013). Taken together, our findings provide molecular (Hancock *et al*. 2011; Alonso‐Blanco *et al*. 2016) and physiological (Blanquart *et al*. 2013; Merilä & Hendry 2014; Monroe *et al*. 2018) evidence that temperature likely causes natural selection in *E. vaginatum*, leading to genetic adaptation and phenotypic change. Climate warming will elicit similar selective pressure to that caused by elevation‐induced temperature change. We thus suggest that warming will cause genetic adaptation in *E. vaginatum*, an abundant plant species of cold northern biomes, adding support to the notion that plants will evolve in the face of rapid climate change (Hairston *et al*. 2005).

We established a reciprocal transplant experiment on one of the elevation gradients that had been well characterised by previous work (Bragazza *et al*. 2012). This allowed us to experimentally test for effects of temperature on plant and carbon cycle processes, and to distinguish between responses acting *via* plasticity and those acting *via* genetic differentiation. We found that plants originating from low elevation invested less in polyamine metabolism and secondary metabolite synthesis than plants adapted to high elevation (Fig. 4a). These pathways link the primary and secondary metabolisms (Forde & Lea 2007; Fraser & Chapple 2011), suggesting that the low elevation population allocated fewer primary metabolites to the production of secondary compounds. This was confirmed by data showing that plants from this population invested differently in the secondary metabolism overall (Fig. 3). Alleviation from cold stress may select for genotypes that produce fewer protective secondary compounds, reflecting a trade‐off between investment in tissue longevity and growth (Díaz *et al*. 2016). We thus suggest that the warmer conditions at low elevation selected against genotypes that divert excess energy and nutrients into cold protection. We found no evidence that high elevation plants alternatively upregulated polyamine metabolism due to greater UVB radiation at their home site, since plant responses to UVB exposure occur rapidly (Radyukina *et al*. 2017) and we observed no plasticity in this pathway for either population (Fig. 4e). Reduced diversion of metabolites away from the primary metabolism likely facilitated the faster growth of the low elevation population (Fig. 4c). In turn, rapid growth is known to accelerate ecosystem respiration (Fig. 4d) through an increase in plant respiration or an associated increase in the activity of soil microbes (Metcalfe *et al*. 2011). Climate warming will lessen cold stress for *E. vaginatum* populations that do not migrate to track the changing climate. Our data suggest that over many generations it will select for genotypes that produce fewer secondary compounds, grow faster and promote greater release of CO_2_ to the atmosphere.

Despite significant source population effects on plant phenotypes, plasticity explained more variation in plant phenotypes overall (57.5% vs. 42.5% explained variance). Plants from both populations displayed a depressed amino acid metabolism when situated at low elevation (Fig. 4e), while also possessing higher concentrations of oxoglutaric acid. Plants use oxoglutaric acid and mineral nitrogen to assemble amino acids (Forde & Lea 2007; Xu *et al*. 2012). Our data suggest that plants at low elevation synthesised fewer amino acids, leading to both a reduced amino acid metabolism and a build‐up of oxoglutaric acid. Warming at these sites has been shown to lower the availability of soil mineral nitrogen by supressing microbial activity and the associated release of ammonium and nitrate from organic matter (Bragazza *et al*. 2012). The warmer conditions at low elevation likely caused plasticity in the *E. vaginatum* metabolism through this mechanism, reducing plant nitrogen availability and the synthesis of amino acids. Such a down‐regulation of nitrogen metabolism at low elevation was also apparent at the leaf scale, where we observed reductions in leaf N, photosynthetic capacity and SLA (Fig. 4f). Photosynthetic rates are tightly coupled to concentrations of the nitrogen‐rich enzyme RuBisCO (Andersson & Backlund 2008). As such, impaired photosynthetic investment was probably also a result of a dampened leaf nitrogen metabolism. In turn, plants at low elevation grew slower (Fig. 4g) and photosynthesised less than plants at high elevation (Fig. 4h), and promoted slower rates of ecosystem respiration. We posit that plasticity in plant nitrogen use was driven by a shift in resource use strategy, not stress, since we found no evidence of stress in any plants (e.g. stomatal conductance, PSII efficiency; Fig. 4f; Appendix S3). For example, plants experiencing nitrogen limitation may invest disproportionately in root biomass to access soil nitrogen (Kant *et al*. 2011). Assuming that warming continues to limit nitrogen availability in this ecosystem and that future adaptation does not occur, these findings together imply that it will cause plasticity in *E. vaginatum* over the lifetime of individual plants that reduces nitrogen metabolism at the molecular scale, impairs photosynthesis at the leaf scale and growth at the plant scale, and slows CO_2_ release to the atmosphere.

Exposure to warmer climate within the lifetime of individual plants caused plasticity that slowed the release of CO_2_ to the atmosphere, whereas over many generations it caused genetic change in plant populations with phenotypes that accelerated CO_2_ release to the atmosphere. We thus show that plant evolutionary responses to warming may oppose those arising through plasticity (Ghalambor *et al*. 2015; Becklin *et al*. 2016), and have the capacity to buffer, or even reverse, short‐term changes to the carbon cycle over timescales of centuries to millennia. These findings demonstrate a clear potential for plant evolution to modify the magnitude and direction of feedbacks from ecosystems to future climate change. The extent to which this will occur depends on whether adaptation keeps pace with rapid climate change (Hairston *et al*. 2005), and whether other factors associated with temperature change, such as plant community interactions, microbial feedbacks to plant growth (Lau & Lennon 2011), or the decoupling of phenologies (Anderson *et al*. 2012; Alexander *et al*. 2015), alternatively become dominant selective forces. We caveat that we measured one species at a single stage of its life cycle, and only quantified ecosystem CO_2_ exchange at a local scale. As such, future studies are needed to test the generality of the responses detected here and their importance for processes of carbon cycling. Nevertheless, we observed similar relationships between elevation, temperature and leaf C and N across three geographically distinct mountain regions (Fig. 1f–h), suggesting that the relationship between temperature and *E. vaginatum* phenotypes is consistent throughout this species’ range. Moreover, temperature causes selection and plasticity in multiple plant species (Hairston *et al*. 2005; Scherling *et al*. 2010; Sardans *et al*. 2011; Kim & Donohue 2013; Anderson & Gezon 2015), raising the possibility that these mechanisms will affect other plant species with consequences for the carbon cycle. This is especially true given that *E. vaginatum* is a perennial sedge that is expected to react slowly to climate change. In summary, our findings illustrate the capacity for plant evolution to modify the immediate effects of climate warming on carbon cycling, and reveal a further mechanism by which plants influence ecosystem responses to climate change.

## Authorship

TW and RDB conceived the study and the experimental design with input from all co‐authors. TW, LB, RDB, CS and SW performed fieldwork. TW did the DNA extractions with support from BF, and the metabolite extractions with support from LF and WW. CS undertook laboratory work relating to leaf level photosynthesis and stoichiometry. DNA sequencing and data preparation were carried out at Edinburgh Genomics, The University of Edinburgh. Metabolite quantification and data validation were undertaken by LF, TW, XS and WW. TW analysed the data and wrote the manuscript in close consultation with RDB and with input from all co‐authors.
