# Supplementary material for: Plastic and genetic responses of a common sedge to warming have contrasting effects on carbon cycle processes
Source: Ecol Lett. 2018 Nov 22;22(1):159–69. doi: 10.1111/ele.13178 (PMC6334510; doi:10.1111/ele.13178)
Supplement: Supplementary file 1 [file ELE-22-159-s001.docx]

**Plastic and genetic responses of a common sedge to warming have contrasting effects on carbon cycle processes**

Tom W. N. Walker, Wolfram Weckwerth, Luca Bragazza, Lena Fragner, Brian G. Forde, Nicholas J. Ostle, Constant Signarbieux, Xiaoliang Sun, Susan E. Ward and Richard D. Bardgett

**SUPPLEMENTARY INFORMATION**

**Appendix S1**

**Supplementary Table S1 | Site information for the three elevation-based climate gradients.** Mean annual temperature (MAT; ºC ± 1 SE) and total annual precipitation (TAP; mm ± 1 SE) were calculated from data sets provided by Provincia Autonoma di Bolzano – Meteo Alto Adige (Italy), Met Office MIDAS data (Scotland; www.midas-data.org.uk) and Meteo Suisse data (Switzerland; [www.meteosuisse.admin.ch](http://www.meteosuisse.admin.ch)).

| **Site** | **Coordinates** | **Elevation (m)** | **MAT (ºC)** | **TAP (mm)** |
| --- | --- | --- | --- | --- |
| *Scotland (Cairngorms)* |  |  |  |  |
| Low | 57º08’N, 03º50’W | 330 | 7.8 ± 0.17 | 952 ± 98 |
| Mid | 57º05’N, 03º51’W | 550 | 6.6 ± 0.17 | 952 ± 98 |
| High | 56º57’N, 03º13’W | 750 | 4.1 ± 0.22 | 1113 ± 141 |
|  |  |  |  |  |
| *Switzerland (Jura/Alps)^1^* |  |  |  |  |
| Low | 46º34’N, 06º10’E | 1000 | 7.4 ± 0.22 | 1051 ± 6 |
| Mid | 46º44’N, 07º22’E | 1400 | 5.7 ± 0.21 | 1025 ± 4 |
| High | 46º36’N, 07º58’E | 1800 | 4.7 ± 0.24 | 1113 ± 2 |
|  |  |  |  |  |
| *Italy (Dolomites)* |  |  |  |  |
| Low | 46º25’N, 11º04’E | 1300 | 7.3 ± 0.06 | 959 ± 3 |
| Mid | 46º25’N, 11º24’E | 1650 | 6.0 ± 0.23 | 629 ± 4 |
| High | 46º21’N, 11º44’E | 1800 | 4.2 ± 0.32 | 1132 ± 8 |

^1^ Site of reciprocal transplant experiment

**Appendix S2**

RAD sequencing and data preparation

Genomic DNA (1 µg) was digested with *PstI*, ligated to an adapter (P1), randomly sheared, filtered to isolate fragments between 300 and 700 bp and then ligated to a second adapter (P2) (Baird *et al.* 2008; Emerson, Merz & Catchen 2010). DNA fragments containing both P1 and P2 adapters were then amplified (Davey *et al.* 2010) and sequenced (HiSeq2000; Illumina, San Diego, USA). We filtered DNA sequence reads in Stacks v1.06 to remove sequencing errors (Catchen *et al.* 2013), following which we built a consensus sequence for each read by aligning reads into clusters of identical sequences (Willing *et al.* 2011). We discarded clusters with a depth of coverage less than eight (Davey *et al.* 2010) or more than two standard deviations above the median stack depth (Emerson *et al.* 2010), and we also removed clusters not present in all populations. For each sample, we aligned remaining reads to consensus sequences (bwa(Li & Durbin 2009) and identified reads containing two versions (“alleles”) of the consensus sequence (GATK Unified Genotyper (McKenna *et al.* 2010).

Primary metabolite analysis and data preparation

Milled samples (5 mg) were extracted with extraction buffer (1150 µl methanol:chloroform:water, 2.5:1:0.5, v/v/v), centrifuged (14,000 *g*, 4 min, 4 ºC), separated into polar and non-polar phases (450 µl ddH_2_O) and dried using a speed-vac concentrator. The polar phase was derivatised (20 µl methoxyamine hydrochloride in 1 ml pyridine, 90 min, 30 ºC) and incubated with MSTFA (30 min, 37 ºC), and the supernatant was analysed using a GC-TOF-MS approach on a gas chromatograph (HP6890, Agilent, Boblingen, Germany) coupled to a TOF mass analyser (Pegasus IV, Leco, St Joseph, USA). Samples were injected twice, once in splitless mode to quantify most primary metabolites and once in split-50 mode to quantify primary metabolites with high concentrations (glucose, fructose, sucrose, quinic acid). We validated data using Leco ChromaTOF software and defined a reference chromatogram from a quality control mix of known primary metabolites. We scrutinised sample chromatograms against the reference chromatogram, quantified peak areas for primary metabolites using fragment ions selected individually for each compound and expressed concentrations on a tissue mass basis (area mg^-1^).

Secondary metabolite analysis and data preparation

We extracted 15 mg milled sample as described above including 1 µg chloramphenicol as an internal standard. After phase separation, dried polar extracts were dissolved in 200 µl solvent (2 % acetonitrile (ACN), 0.1 % formic acid (FA)), centrifuged and injected (20 µl) into a LC system (Dionex Ultimate 3000 XRS, Thermo Fisher, Schwerte, Germany) coupled to an Orbitrap mass spectrometer (FTMS, LTQ-Orbitrap XL, Thermo Fisher, Schwerte, Germany). Compound separation was performed on a C18 column (flow rate of 100 µl min^-1^, from 1 % solvent B (90 % ACN, 0.1 % FA) isocratic for 2 min to 90 % solvent B isocratic over 40.5 min and held isocratic for 4.5 min). Accurate masses of ions for mass formula were determined with the Orbitrap FTMS mass analyser using the lock mass option and a mass resolution of 30,000 in full scan mode. We then calculated atomic ratios in secondary metabolites following(Doerfler *et al.* 2014). Specifically, we selected the most abundant ions (m/z features, intensity threshold 5e5), calculated sum formula and inferred their chemical formulae (m/z error < 5 ppm, N/C ≤ 1.3, O/C ≤ 1.2, H/C = 0.33-3.1; (Kind & Fiehn 2007).

Climate-induced genetic adaptation & local adaptation

We used the reciprocal transplant experiment to test for the presence of standing genetic variation in *E. vaginatum* populations (Merilä & Hendry 2014; Monroe *et al.* 2018). This was achieved using an RDA with forward selection to test for source population effects on plant phenotypes after accounting for planting elevation effects. We expressed plant phenotypes as a multivariate data matrix including metabolites expressed at the pathway level (amino acid metabolism, carbohydrate metabolism, TCA cycle, polyamine metabolism, secondary metabolite synthesis, other), leaf traits (leaf C, leaf N, SLA, *A*_max_, *g*_s_ and PSII efficiency) and plant growth (height increase, biomass production). While explicitly testing for local adaptation was not possible, we used the same RDA approach to examine whether populations displayed consistent phenotypes at their home versus away site using an RDA on the same data matrix. This was done by comparing the phenotype data matrix between home and away populations after accounting for source population and planting elevation effects (Blanquart *et al.* 2013).

Adaptation and plasticity effects on phenotypes

We tested for effects of population, elevation and their interaction on the activity of the leaf primary metabolism between populations and elevations using standardized linear mixed effects models (LMEs) including compound nested within plant as a random intercept term. We also compared primary metabolisms using a PERMANOVA by Euclidean distance, alongside standardised generalised least squares (GLS) models on the first two components of a principal component analysis. We tested for effects of source population, elevation and their interaction on individual primary metabolite concentrations using standardised GLS models, and on leaf primary metabolite pathways using standardised LMEs including compound name as a random intercept term. For the secondary metabolism, we tested whether relationships between total atom counts of carbon, hydrogen, oxygen and nitrogen differed between populations using LMEs, including planting elevation as a random intercept term (see Appendix S5). Standardised LMEs were also used to test for effects of source population (within planting elevation) and planting elevation (within source population) on A*_max_* and g*_s_* by including planting elevation and source population as random intercept terms, respectively. We tested for effects of source population, planting elevation and their interaction on SLA, PSII efficiency, leaf C, N and plant growth using standardised GLS models. We tested for effects of source population, planting elevation and their interaction on NEE, gross photosynthesis and ER using standardised LME models, including sampling date as a random intercept term. GLS and LME models were scrutinised against test assumptions and, where necessary, optimised to account for non-normality of residuals or unequal variance between explanatory variables. Statistical significance (*P* < 0.05) was determined using single term deletions coupled to likelihood ratio tests, and was reported alongside likelihood ratios and numerator and residual degrees of freedom for fixed effects.

**References**

Baird, N.A., Etter, P.D., Atwood, T.S., Currey, M.C., Shiver, A.L., Lewis, Z.A., Selker, E.U., Cresko, W.A. & Johnson, E.A. (2008) Rapid SNP discovery and genetic mapping using sequenced RAD markers. *PLoS ONE*, **3**, e3376.

Blanquart, F., Kaltz, O., Nuismer, S.L. & Gandon, S. (2013) A practical guide to measuring local adaptation (ed D Ebert). *Ecology Letters*, **16**, 1195–1205.

Catchen, J., Hohenlohe, P.A., Bassham, S., Amores, A. & Cresko, W.A. (2013) Stacks: An analysis tool set for population genomics. *Molecular ecology*, **22**, 3124–3140.

Davey, J.W., Davey, J.L., Blaxter, M.L. & Blaxter, M.W. (2010) RADSeq: next-generation population genetics. *Briefings in functional genomics*, **9**, 416–423.

Doerfler, H., Sun, X., Wang, L., Engelmeier, D., Lyon, D. & Weckwerth, W. (2014) mzGroupAnalyzer-Predicting Pathways and Novel Chemical Structures from Untargeted High-Throughput Metabolomics Data. *PLoS ONE*, **9**, e96188–.

Emerson, K.J., Merz, C.R. & Catchen, J.M. (2010) Resolving postglacial phylogeography using high-throughput sequencing. *Proceedings of the National Academy of Sciences*, **107**, 16196–16200.

Kind, T. & Fiehn, O. (2007) Seven Golden Rules for heuristic filtering of molecular formulas obtained by accurate mass spectrometry. *BMC Bioinformatics*, **8**, 105.

Li, H. & Durbin, R. (2009) Fast and accurate short read alignment with Burrows – Wheeler transform. *Bioinformatics*, **25**, 1754–1760.

McKenna, A., Hanna, M., Banks, E., Sivachenko, A., Cibulskis, K., Kernystky, A., Garimella, K., Altshuler, D., Gabriel, S., Daly, M. & DePristo, M.A. (2010) The Genome Analysis Toolkit: A MapReduce framework for analyzing next-generation DNA sequencing data. *Genome Research*, **20**, 1297–1393.

Merilä, J. & Hendry, A.P. (2014) Climate change, adaptation, and phenotypic plasticity: the problem and the evidence. *Evolutionary Applications*, **7**, 1–14.

Monroe, J.G., Markman, D.W., Beck, W.S., Felton, A.J., Vahsen, M.L. & Pressler, Y. (2018) Ecoevolutionary Dynamics of Carbon Cycling in the Anthropocene. *Trends in Ecology and Evolution*, **33**, 213–225.

Willing, E.-M., Hoffmann, M., Klein, J.D., Weigel, D. & Dreyer, C. (2011) Paired-end RAD-seq for de novo assembly and marker design without available reference. *Bioinformatics*, **27**, 2187–2193.

**Appendix S3**

No physiological stress in transplanted *E. vaginatum* tussocks

We explored whether disturbance (i.e. removal and replanting) of *E. vaginatum* individuals created a stress to *E. vaginatum* plants that could modify its responses to temperature change. We measured leaf primary metabolite concentrations (expressed at the metabolite pathway level), specific leaf area (SLA), leaf photosynthetic capacity (A_max_), leaf stomatal conductance (g_s_), leaf maximum PSII efficiency (F_v_/F_m_) and plant growth on undisturbed plants at each planting elevation (n = 4 for physiology, n = 10 for plant growth). We compared response variables between this undisturbed group and the disturbed plants originating from the same site using linear mixed effects models, including a random intercept term for planting site. We found that *E. vaginatum* metabolism, SLA, leaf photosynthetic capacity, stomatal conductance, F_v_/F_m_ and growth were all unaffected by disturbance (Supplementary Table S2; *P* > 0.05 in all cases), revealing that *E. vaginatum* individuals were not physiologically stressed by transplantation.

**Supplementary Table S2 | Statistics for disturbance effect on *E. vaginatum* physiology.** Significant *P*-values (*P* < 0.05) are displayed in bold. Degrees of freedom (df) are numerator and residual degrees of freedom for fixed effects.

| **Response** | **Disturbance effect** | | |
| --- | --- | --- | --- |
|  | LR | df | *P* |
| Leaf metabolism |  |  |  |
| Amino acid metabolism | 1.65 | 1,73 | 0.1986 |
| Carbohydrate metabolism | 0.84 | 1,73 | 0.3599 |
| Polyamine metabolism | 0.24 | 1,73 | 0.6257 |
| 2º metabolite synthesis | 0.01 | 1,73 | 0.9346 |
|  |  |  |  |
| Plant physiology |  |  |  |
| Maximum PSII efficiency (F_v_/F_m_) | 0.01 | 1,12 | 0.9278 |
| Stomatal conductance (g*_s_*) | 1.18 | 1,12 | 0.2779 |
| Photosynthetic capacity (A*_max_*) | 0.01 | 1,12 | 0.9163 |
| Specific leaf area (SLA) | 1.80 | 1,12 | 0.1803 |
| Plant growth | 0.00 | 1,35 | 0.9765 |

**Appendix S4**


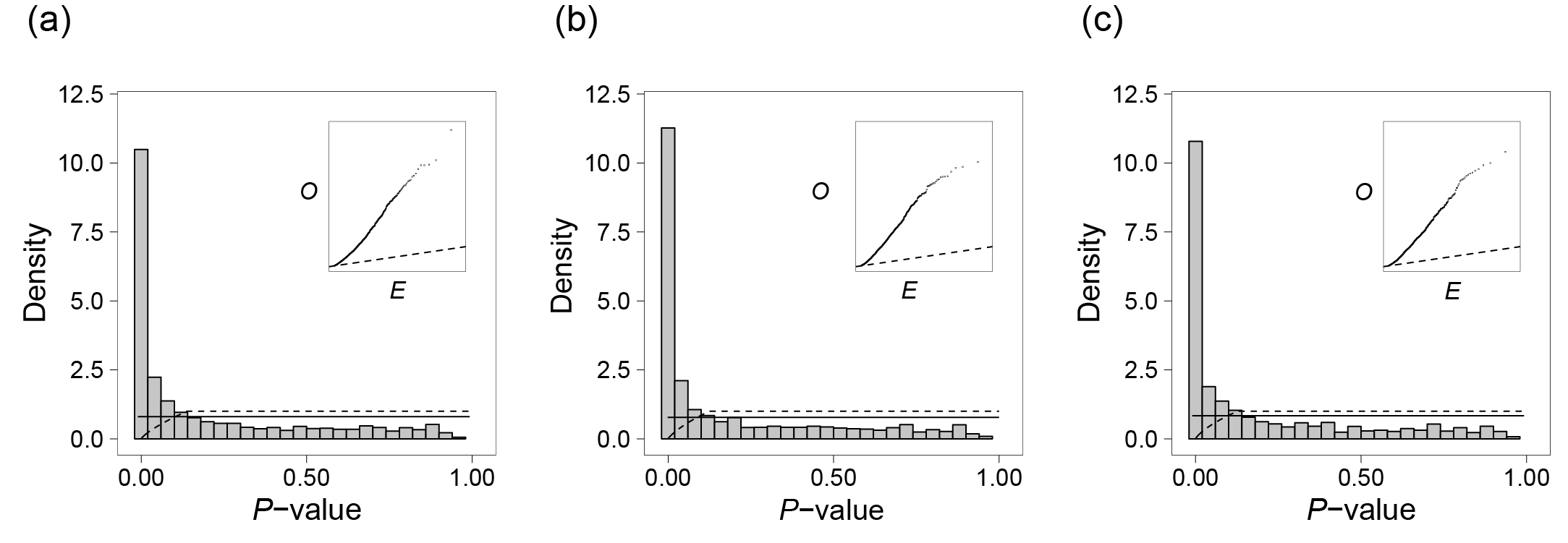


**Supplementary Figure S1 | Significance of allele frequency changes between elevations.** Histograms showing the distributions of *P-*values generated by pairwise Cochran-Mantel-Haenszel tests of allele frequency changes between (a) high-low, (b) high-mid and (c) mid-low elevations, considering all identified genetic mutations present in three replicate gradients. Significant *P*-values (*P* < 0.05) are considered truly significant if their frequency is greater than both local (dotted line) and overall (solid line) false discovery rates. Statistical significance is further supported if plots of observed (*O*) versus expected (*E*) -log_10_ transformed *P-*values (inlays; points) show a positive deviation of transformed *P*-values from a 1:1 line (dotted line).

**Appendix S5**

**Supplementary Table S3 | Statistics for mountain gradients.** Significant *P*-values (*P* < 0.05) are displayed in bold. Degrees of freedom (df) are numerator and residual degrees of freedom for fixed effects.

| **Response** | **Elevation/Population** | | |  | **Gradient (site)** | | |  | **Elevation ✕ Gradient** | | |
| --- | --- | --- | --- | --- | --- | --- | --- | --- | --- | --- | --- |
|  | LR | df | *P* |  | LR | df | *P* |  | LR | df | *P* |
| Mean annual temperature (MAT) | **28.71** | **1,5** | **< 0.0001** |  | 1.77 | 2,5 | 0.4133 |  | 3.82 | 2,3 | 0.1482 |
| Total annual precipitation (TAP) | 1.87 | 1,5 | 0.1716 |  | 2.52 | 2,5 | 0.2837 |  | 0.17 | 2,3 | 0.9169 |
| Global radiation | 1.27 | 1,34 | 0.2589 |  | 2.26 | 2,34 | 0.3230 |  | **34.80** | **2,32** | **< 0.0001** |

**Supplementary Table S4 | Summary of statistics for reciprocal transplant.** Significant *P*-values (*P* < 0.05) are displayed in bold. Degrees of freedom (df) are numerator and residual degrees of freedom for fixed effects. Arrows indicate an increase or decrease illustrating a higher value for the low elevation source population or planting elevation.

| **Response** | **Source population** | | |  | **Planting elevation** | | |  | **Source ✕ Elevation** | | | |
| --- | --- | --- | --- | --- | --- | --- | --- | --- | --- | --- | --- | --- |
|  | LR | df | *P* |  | LR | df | *P* |  | LR | df | | *P* |
| Leaf metabolism |  |  |  |  |  |  |  |  |  |  | |  |
| 1º metabolism activity | **6.90** | **1,17** | **0.0086** | ↓ | 0.79 | 1,17 | 0.3740 |  | 0.01 | 1,15 | | 0.9268 |
| 1º metabolite PC1 scores | **10.35** | **1,17** | **0.0013** | ↑ | 1.01 | 1,17 | 0.3159 |  | 0.34 | 1,15 | | 0.5608 |
| 1º metabolite PC2 scores | 0.42 | 1,17 | 0.5161 |  | **22.89** | **1,17** | **< 0.0001** | ↑ | 0.61 | 1,15 | | 0.4341 |
| Amino acid metabolism | **25.27** | **1,341** | **< 0.0001** | ↓ | **39.32** | **1,341** | **< 0.0001** | ↓ | 0.05 | 1,339 | | 0.8253 |
| Carbohydrate metabolism | **31.32** | **1,341** | **< 0.0001** | ↓ | 1.72 | 1,341 | 0.1898 |  | 0.15 | 1,339 | | 0.7021 |
| Polyamine metabolism | **6.94** | **1,341** | **0.0084** | ↓ | 0.10 | 1,341 | 0.7514 |  | 0.12 | 1,339 | | 0.7283 |
| 2º metabolite synthesis | **21.03** | **1,341** | **< 0.0001** | ↓ | 3.05 | 1,341 | 0.0808 |  | 0.13 | 1,339 | | 0.7193 |
|  |  |  |  |  |  |  |  |  |  |  | |  |
| Plant physiology |  |  |  |  |  |  |  |  |  |  | |  |
| Leaf N content | 0.01 | 1,14 | 0.9337 |  | **9.77** | **1,14** | **0.0018** | ↓ | 2.74 | 1,12 | | 0.0980 |
| Leaf C content | 0.85 | 1,14 | 0.3558 |  | 1.42 | 1,14 | 0.2328 |  | 1.48 | 1,12 | | 0.2244 |
| Leaf C:N | 1.08 | 1,6 | 0.2999 |  | **10.10** | **1,6** | **0.0015** | ↑ | 2.17 | | 1,7 | 0.1411 |
| PSII efficiency (F_v_/F_m_) | 0.03 | 1,14 | 0.8607 |  | 0.13 | 1,14 | 0.7166 |  | 0.64 | 1,12 | | 0.4248 |
| Stomatal conductance (g*_s_*) | 1.73 | 1,12 | 0.1890 |  | 1.96 | 1,12 | 0.1610 |  | - | - | | - |
| Photosynthetic capacity (A*_max_*) | 1.62 | 1,12 | 0.2024 |  | **6.03** | **1,12** | **0.0140** | ↓ | - | - | | - |
| Specific leaf area (SLA) | **14.84** | **1,14** | **0.0271** |  | **19.22** | **1,14** | **0.0023** | ↓ | 0.01 | 1,12 | | 0.9220 |
| Plant growth | **5.28** | **1,37** | **0.0126** | ↑ | **11.19** | **1,37** | **0.0008** | ↓ | 0.13 | 1,35 | | 0.7200 |
|  |  |  |  |  |  |  |  |  |  |  | |  |
| Ecosystem CO_2_ fluxes |  |  |  |  |  |  |  |  |  |  | |  |
| Ecosystem respiration (ER) | **6.30** | **1,72** | **0.0120** | ↑ | **13.57** | **1,72** | **0.0002** | ↓ | 0.03 | 1,70 | | 0.8537 |
| Gross photosynthesis | 1.18 | 1,71 | 0.2772 |  | **4.37** | **1,71** | **0.0365** | ↓ | 0.01 | 1,69 | | 0.9342 |
| Net CO_2_ exchange (NEE) | 0.09 | 1,71 | 0.7612 |  | 0.28 | 1,71 | 0.5985 |  | 0.11 | 1,69 | | 0.7450 |

**Appendix S6**


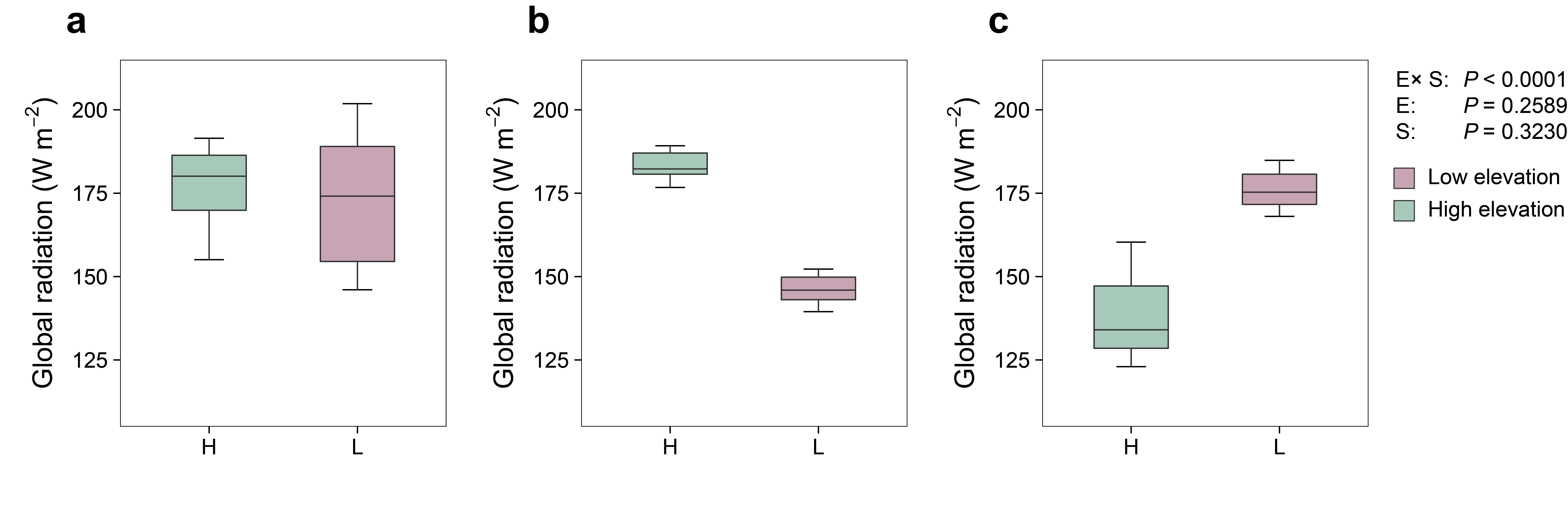


**Supplementary Figure S2 | Elevation effects on global radiation.** Differences in global radiation, comprising solar and diffuse radiation, between low (red) and high (green) elevation sites at the (a) Cairngorms, (b) Alps/Jura and (c) Dolomites gradients. *P-*values indicate the significance (*P* < 0.05) of differences between elevations (E), gradients (S) and their interaction (E × S), as determined by generalised least squares models. Elevation effects on global radiation varied significantly between gradients, illustrating no consistent effect of elevation on global radiation when considering all gradients simultaneously.

**Appendix S7**

**
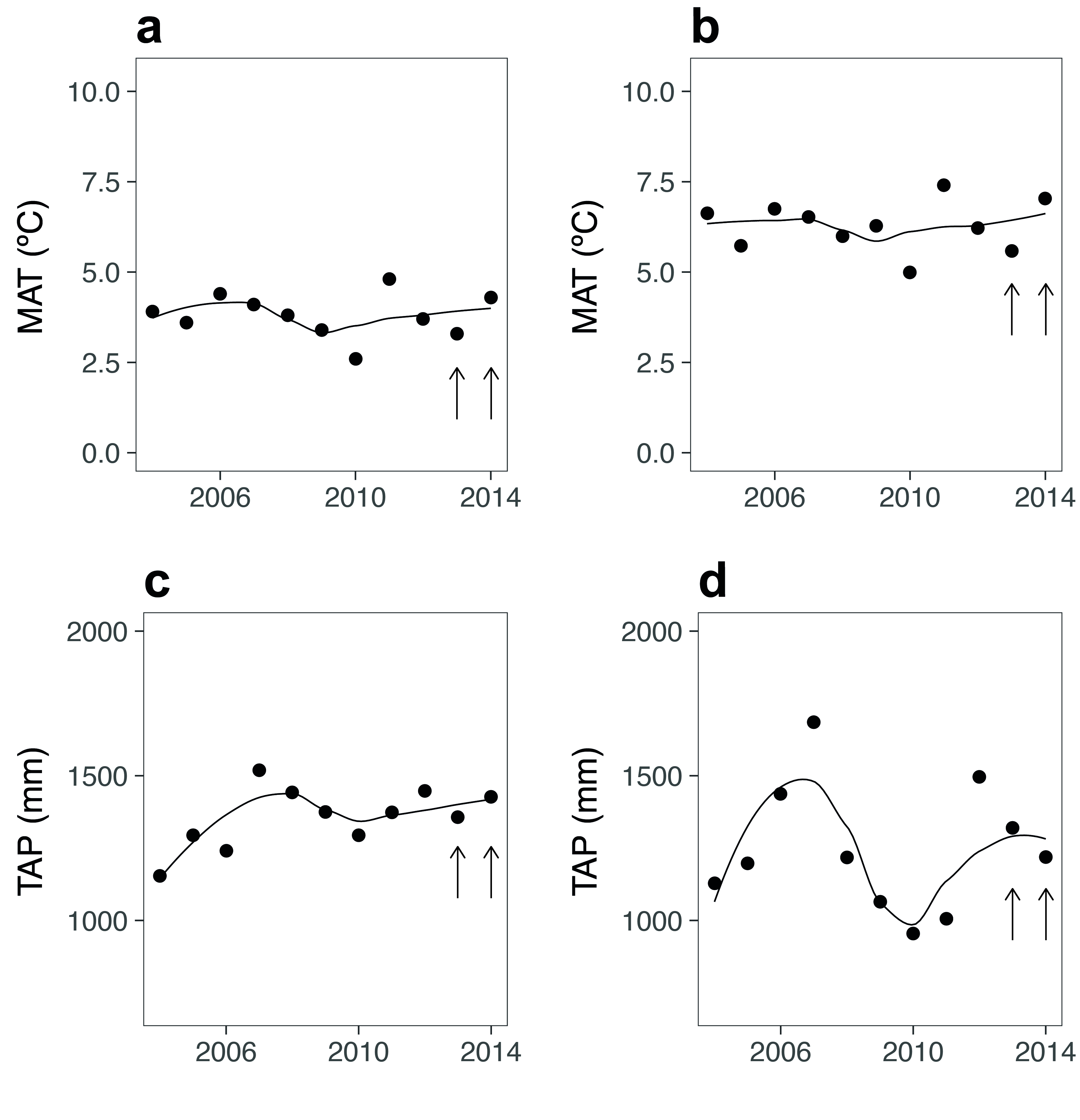
**

**Supplementary Figure S3 | Interannual climate variation on the Alps gradient.** Time series showing the variability in (a,b) mean annual temperature (MAT; ºC) and (c,d) total annual precipitation (TAP; mm) at (a,c) high and (b,d) low elevation sites of the reciprocal transplant experiment between 2004 and 2014. Lines represent the output of a loess smoothing function, and the two study years are indicated by arrows underneath the points.

**Appendix S8**

**Supplementary Table S5 | Significant primary metabolite changes under warming.** Primary metabolites displaying significant (*P* < 0.05) increases (↑) or decreases (↓) in concentrations following adaptation (source population effects) and plasticity (planting elevation effects). Significance was determined using likelihood ratio tests between models including and excluding explanatory variables (Methods).

| **Compound** | **Effect** |  |
| --- | --- | --- |
|  | Source population | Planting elevation |
| *Amino acid metabolism* |  |  |
| Aspartic acid | ↓ |  |
| Glutamic acid | ↓ | ↓ |
| Glycine |  | ↓ |
| Isoleucine |  | ↓ |
| Leucine |  | ↓ |
| Pyrolglutamic acid | ↓ | ↓ |
| Serine |  | ↓ |
| Threonine |  | ↓ |
| Tryptophan | ↓ | ↑ |
| Valine |  | ↓ |
| Glyceric acid | ↓ |  |
|  |  |  |
| *Carbohydrate metabolism* |  |  |
| CHO ((RI 3052) | ↓ |  |
| CHO (RI 2227) |  | ↑ |
| CHO (RI 2808) | ↓ |  |
| CHO (RI 3077) | ↓ | ↑ |
| CHO (RI 3214) | ↓ |  |
| Disaccharide (RI 2810) | ↓ |  |
| Erythrose-threose |  | ↑ |
| Glucose | ↓ |  |
| HBG | ↓ | ↓ |
| Polysaccharide (RI 3510) | ↓ |  |
| Threonic-erythreonic acid |  | ↓ |
|  |  |  |
| *Polyamine metabolism* |  |  |
| Nicotinic acid | ↓ |  |
| Putrescine |  | ↓ |
| Spermidine | ↓ | ↑ |
|  |  |  |
| *Secondary metabolite synthesis* |  |  |
| Caffeoylquinic acid | ↓ |  |
| Chlorogenic acid | ↓ |  |
| Shikimic acid | ↓ |  |
|  |  |  |
| *Other* |  |  |
| Oxoglutaric acid | ↓ | ↑ |
| Myo-inositol |  | ↓ |
| Ascorbic acid | ↑ |  |
| Pentonic acid | ↓ |  |
| Glycolic acid | ↓ | ↓ |
| Lactic acid |  | ↓ |
| Phosphoric acid |  | ↓ |
| Sugar phosphate (RI 2375) | ↓ | ↓ |
